# Isoniazid-induced Lupus: When the Cure Can Be Lethal

**DOI:** 10.7759/cureus.7311

**Published:** 2020-03-18

**Authors:** Ana Cerqueira, Tiago Seco, David Paiva, Helio Martins, Jorge Cotter

**Affiliations:** 1 Internal Medicine, Hospital Senhora Da Oliveira, Guimarães, PRT

**Keywords:** drug-induced-lupus, isoniazid, idiosyncratic reaction

## Abstract

A 50-year-old female with a past medical history of bone tuberculosis diagnosed nine months ago was admitted in our infirmary for persistent fever with no evident cause. The patient was treated with isoniazid, rifampicin, pyrazinamide, and ethambutol for seven months and for the past two months, she was taking isoniazid and rifampicin. She went to our emergency room (ER) for back pain and fever that she had been experiencing for the last month. She was admitted with suspicion of disseminated tuberculosis that was never confirmed. Physical examination was unremarkable. Blood tests showed an elevation of inflammation parameters. A computed tomography (CT) scan of the chest showed a mild pleural effusion. She remained with fever during the three weeks in the infirmary while undergoing many other studies that were all negative. The back pain would change sides, and three consecutive thoracic radiographies showed a small-sized pleural effusion that was either predominantly right-sided or left-sided. Several differential diagnoses were considered in the process, namely an active infection, neoplasia, or autoimmune disease. The search for circulating lupus anticoagulant was positive. Antinuclear antibodies (ANA) were positive and the anti-histone antibody was strongly positive. At this point, we suspected a drug-induced lupus diagnosis, and isoniazid was discontinued. Following discontinuation of isoniazid, back pain and fever subsided and patient was discharged after one week. This case is a diagnostic challenge because of the rarity and symptom severity of isoniazid-induced lupus. Isoniazid rarely induced this lupus-like syndrome, with an incidence of considerably less than 1%.

## Introduction

Drug-induced lupus erythematosus (DILE) is an idiosyncratic reaction where a drug exposure leads to the development of systemic lupus erythematosus (SLE) like clinical features, without pre-existing lupus [1]. It is an autoimmune phenomenon that has been accepted as a side effect of therapy with dozens of drugs since its first description in association with sulfadiazine in 1945. Drug-induced lupus may develop a few weeks to several months after starting the offending drug, and therefore, it usually represents a very difficult diagnosis. Furthermore, it is not possible to differentiate DILE from SLE based on clinical features alone. Although DILE tends to be milder, renal or central nervous system involvement, vasculitis, leukopenia, serositis, and pericarditis have rarely been reported [2]. Laboratory evaluation is crucial, especially regarding autoantibodies evaluation. In both diseases, it is known that autoimmune analysis frequently reveals a positive antinuclear antibodies (ANA), usually in a homogenous pattern. Anti-histone antibodies are present in 75% of cases of DILE, but not in SLE. Up to 25% of patients taking isoniazid have detectable ANA titres, but only 1% develop systemic DILE [3]. Constant pharmacovigilance is crucial for prompt diagnosis, but the mainstay of treatment is recognition and discontinuation of the offending drug [4].

Herein we present a case of a 50-year-old female that developed isoniazid-induced lupus after nine months of treatment of bone tuberculosis. The clinical clues were persistent fever and serositis.

## Case presentation

A 50-year-old Caucasian female presented to our emergency room (ER) reporting fever and back pain for the past one month. The patient had been diagnosed with bone tuberculosis nine months before and was treated with isoniazid, rifampicin, pyrazinamide and ethambutol during the first seven months and with isoniazid and rifampicin since then.

**Figure 1:**
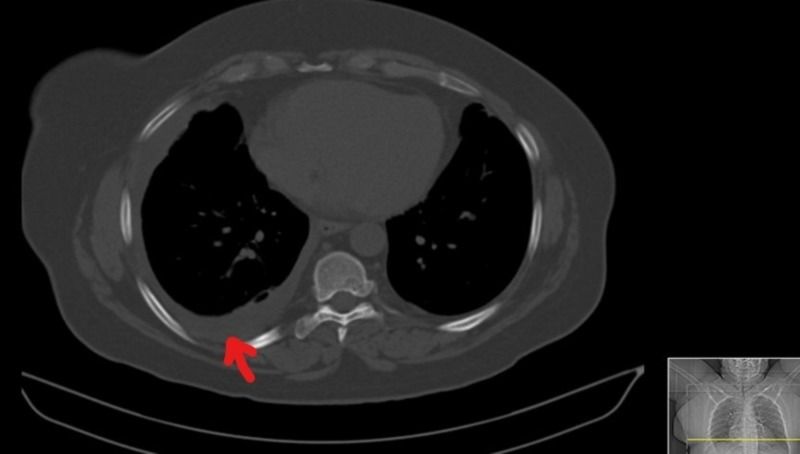
Thoracic computed tomography (CT) scan from day 1

Physical examination was unremarkable. Blood tests showed a sedimentation rate (SR) of 64 mm (normal 0-19) and C-reactive protein (CRP) of 310 mg/L (normal < 2.9). A computed tomography (CT) scan of the chest showed only a small right pleural effusion (Figure 1).

**Figure 2:**
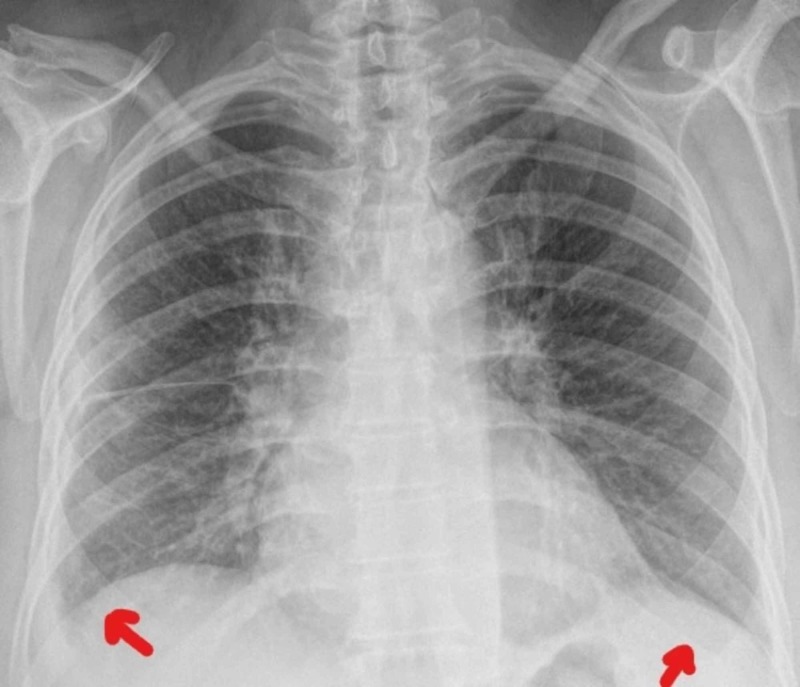
Thoracic radiography from day 19

**Figure 3:**
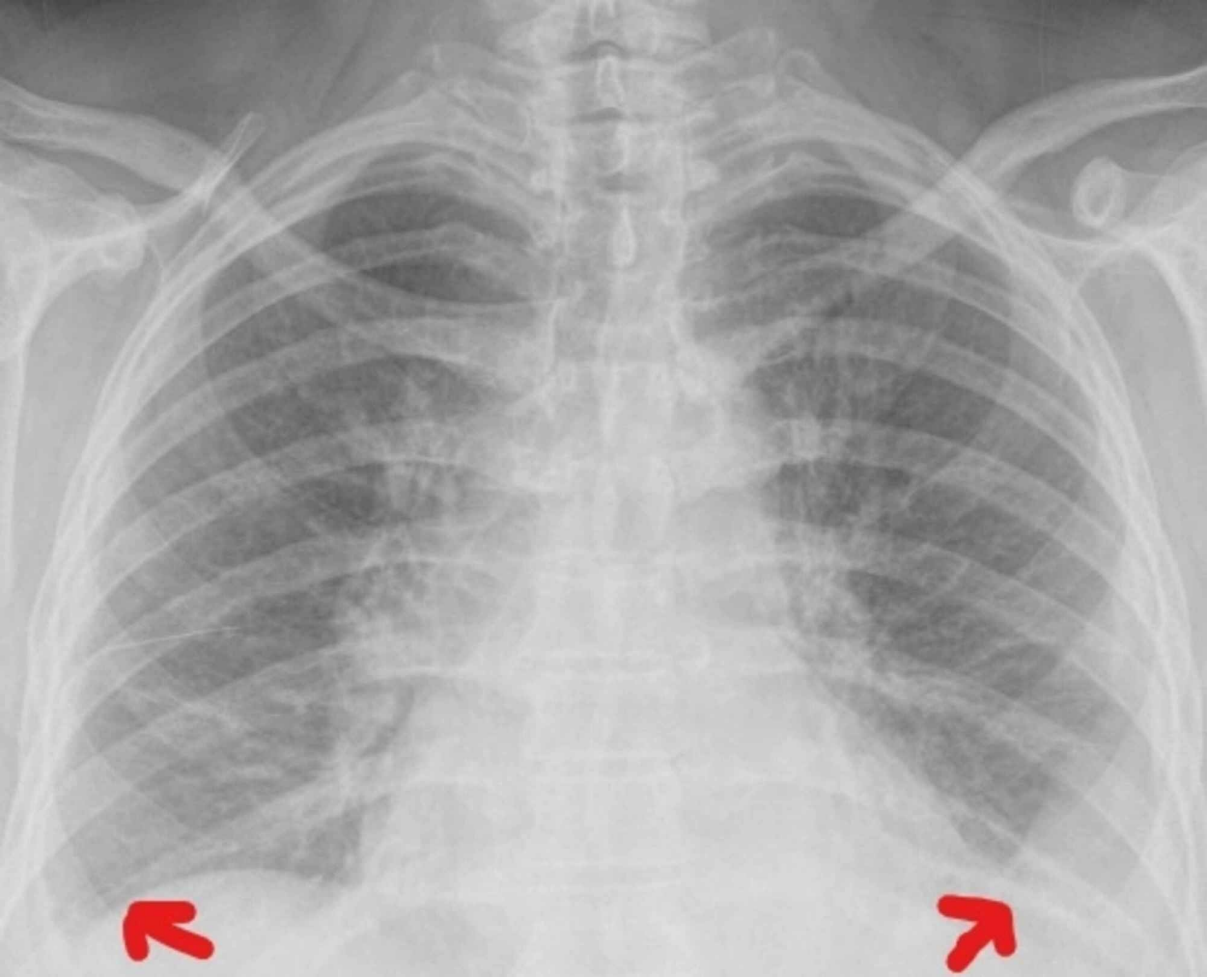
Thoracic radiography from day 11

**Figure 4:**
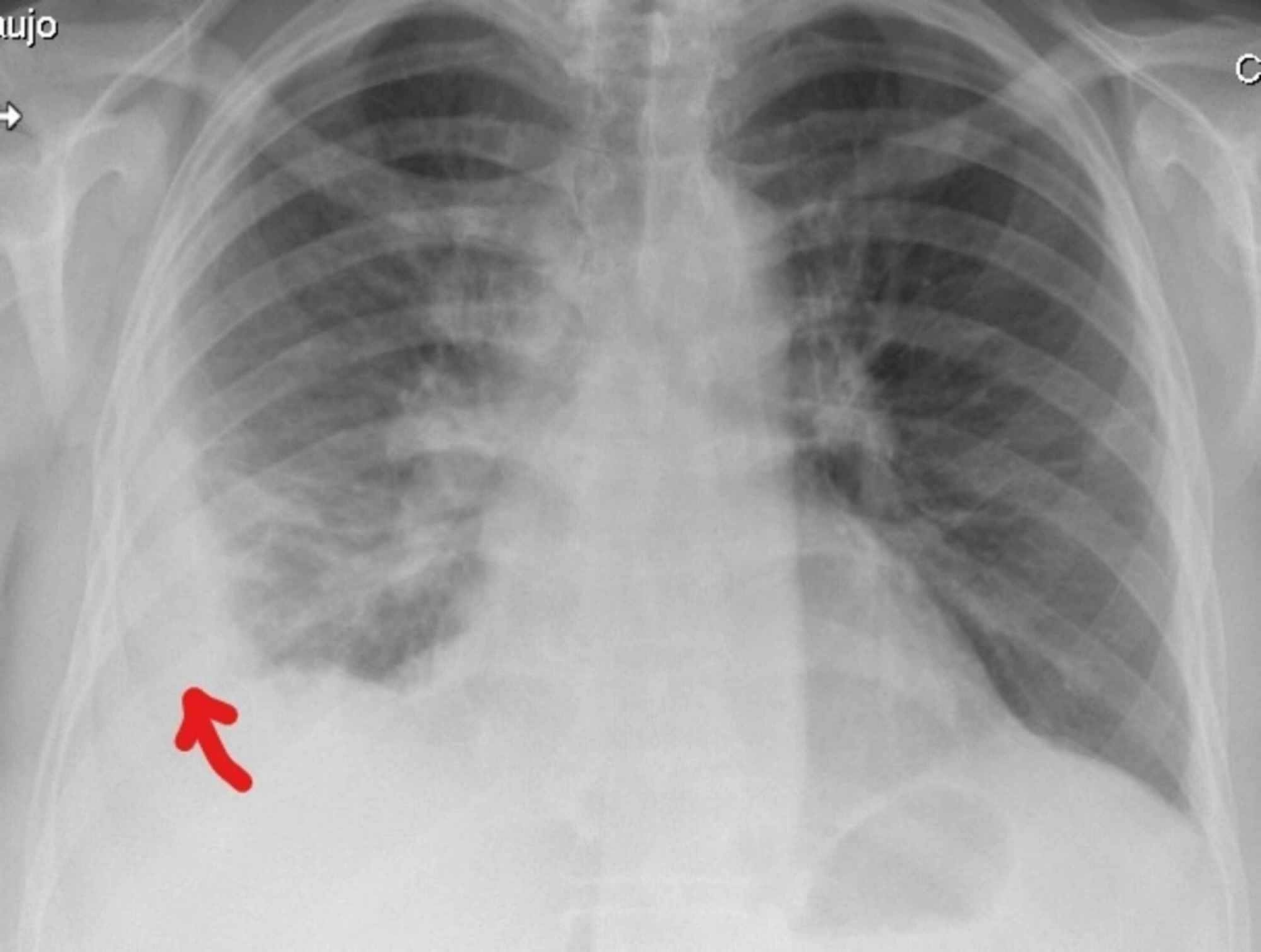
Thoracic radiography from day 5

She was admitted to the Internal Medicine department with a suspicion of a disseminated tuberculosis. She remained with fever during almost three weeks and all the other studies were unremarkable. The physical examination remained unchanged and the blood results showed CRP values ​​increasing with a maximum of 380 mg/L. The initial hypothesis of disseminated tuberculosis was never confirmed. The back pain fluctuated from right side to left side, and three consecutive thoracic radiographies showed a small-sized pleural effusion that was either predominantly right-sided or left-sided, suggesting serositis (Figures 2-4). Throughout the whole hospitalization, no diagnostic thoracentesis was done because of small pleural effusion.

The search for circulating lupus anticoagulant was positive. ANA was positive at a 1/1280 titer (homogenous standard) and the anti-histone antibody was strongly positive (immunoglobulin M (IgM)). At this point, DILE was suspected, in which case, isoniazid was most probably involved. Isoniazid was thus suspended resulting in resolution of all symptoms and clinical findings. The patient remained asymptomatic and was discharged after one week of drug suspension with a CPR of 42 mg/L. She was reevaluated as an outpatient at 1, 3 and 6 months, one and two years after discharge. She remained asymptomatic without evidence of recrudescence or any other autoimmune disorder. During follow-up, inflammation markers, namely sinus rhythm (SR) and CRP, have been persistently within normal values, ANA had become negative while anti-Smith (anti-Sm), anti-ribonucleoprotein (anti-RNP), antineutrophil cytoplasmic antibodies (ANCA), and anticardiolipin have remained negative. Anti-histone antibodies only became negative after two years.

## Discussion

DILE is an autoimmune process whose clinical, histological, and immunological features are similar to those in SLE but usually resolves completely with the withdrawal of the offending drug [1]. The lupus phenomenon is an idiosyncratic reaction and represents 10% of all cases of lupus [5]. The pathophysiology of DILE remains unclear, although it appears that different mechanisms are responsible for the autoimmune response by different lupus-inducing drugs. The human leukocyte antigen (HLA)-DR4, HLA-DR0301, and complement C4 were identified as genetic risk factors [6]. It is known that slow acetylators with genetic deficiency of N-acetyltransferase are also at a higher risk of DILE. In this case, the inhibition of DNA methylation of CD4+ T cells is responsible for overstimulating autoantibody production by interaction with self-class II major histocompatibility complex (MHC) molecules on B-cells and consequent induction of apoptosis in macrophages. The dying cells release the highly antigenic apoptotic chromatin. This autoantibody production, as well as the release of the antigenic macrophage chromatin, is thought to contribute to the development of lupus-like autoimmunity [5]. DILE is an exclusion diagnosis, nonetheless, five criteria are necessary: ​​clinical features must be absent before the administration of the drug; the drug must be known to be able to cause a lupus-like syndrome; symptoms must be reversible upon discontinuation of the molecule in question; ANA positivity plus at least one clinical feature of SLE; and the reappearance of pathological manifestations if ever the drug is reintroduced, which, for obvious reasons, is never purposely tested. Isoniazid is classified as a major first-line anti-tuberculosis drug and isoniazid-induced lupus occurs in 1% of cases, although 25% of patients receiving treatment with isoniazid have positive ANA [2]. Isoniazid-induced lupus syndrome normally occurs with doses of at least 300 mg/day with a treatment duration of six months to two years. In our patient, these conditions were fulfilled, but it may not always be so. Jguirim et al. reported two cases of isoniazid-induced lupus in young women; after three months of treatment to lymph node tuberculosis, one of them presented with polyarthralgia, a pruritic maculopapular rash in the photo-exposed regions and diffuse bald patches in the scalp; the other one presented with tenosynovitis in the right hand and arthritis in the left ankle after one month of exposure to isoniazid. Both had leukopenia and anemia [7].

The clinical features of DILE are usually mild compared to SLE, and they are dominated by articular and muscle damage in 50% to 90% of cases. Cutaneous signs are present in 25% to 53% of cases with rare visceral, neurological and renal involvement. Anemia, leukopenia and thrombocytopenia are rarely seen. The level of ANA is conventionally low and the presence of antihistone antibodies is suggestive but not pathognomonic [8]. Luwanda et al. also reported a isoniazid-induced lupus characterized mainly by cutaneous manifestations. In this case, a 40-year-old human immunodeficiency virus (HIV)-positive female presented with an erythematous macular eruption involving the malar and periorbital area, the forehead, and neck after six weeks of treatment with co-formulated tenofovir/lamivudine/efavirenz plus isoniazid prophylaxis. In this patient, ANA and antihistone antibodies were negative. Considering the possible role of isoniazid led to its suspension, allowing for the diagnosis and clinical resolution [9]. Although systemic glucocorticoids may be required to treat severe cases of serositis, the identification and discontinuation of the culprit drug is the most important factor in the prognosis of the disease. ANA often persist positive after the resolution of symptoms and physical findings, and in some patients, may persist for a year or longer. Anti-histone antibodies are present in more than 95% of patients overall and is a hallmark for DILE. In our patient, the antihistone antibody remained positive for almost two years. Last year, Vaghela et al. reported the case of a 14-year-old female with an isoniazid induced lupus after being treated for one week for meningitis tuberculosis. Although she had a past medical history of an allergic reaction to isoniazid (cutaneous presentation), this time the patient complained of fever, common cold, muscle weakness, joint pain, convulsion, and multiple oral ulcers. Sodium valproate was then prescribed for the convulsions and after 15 days without isoniazid, she improved dramatically [10].

In therapeutic schemes as complex as the ones used with tuberculosis or HIV, awareness of the possibility of DILE in the right clinical setting would prompt early recognition and management of this condition [11]. There is no doubt that our patient developed an isoniazid induced lupus, but the combination of persistent fever and serositis remains an uncommon presentation.

## Conclusions

Anti-tuberculosis drugs are responsible for frequent side effects. Although mainly minor, sometimes severe reactions are found. Isoniazid-induced lupus should be considered in the setting of clinical and immunological findings suggestive of lupus. This case presented a challenge due to its atypical presentation and severity of symptoms. The association of persistent fever and serositis had yet to be reported in this setting. The identification along with discontinuation of the offending drug results in the resolution of symptoms.
